# Traditional Vietnamese Medicine Containing Garlic Extract for Patients With Non-severe COVID-19: A Phase-II, Double-Blind, Randomized Controlled Trial

**DOI:** 10.7759/cureus.42484

**Published:** 2023-07-26

**Authors:** Tran Van Giang, Le Nguyen Minh Hoa, Tran Thi Hien, Quach Duy Cuong, Nguyen Trung Cap, Nguyen Lam Vuong, Pham Ngoc Thach

**Affiliations:** 1 Department of Viral and Parasitic Diseases, National Hospital for Tropical Diseases, Hanoi, VNM; 2 Department of Infectious Diseases, Hanoi Medical University, Hanoi, VNM; 3 Department of Microbiology and Molecular Biology, National Hospital for Tropical Diseases, Hanoi, VNM; 4 Emergency Department, National Hospital for Tropical Diseases, Hanoi, VNM; 5 Department of Medical Statistics and Informatics, Faculty of Public Health, University of Medicine and Pharmacy at Ho Chi Minh City, Ho Chi Minh City, VNM

**Keywords:** sars-cov-2, covid-19, allium sativum, garlic, herbal, traditional medicine

## Abstract

Background

Coronavirus disease 2019 (COVID-19) is still ongoing with the omicron variant. Low-cost, effective treatments are still needed, particularly in low-to-middle-income countries. This study assessed the safety and efficacy of TD0068, an herbal medicine developed from mainly garlic, for patients with non-severe COVID-19.

Methods

This is a phase-II, double-blind, randomized controlled trial to compare oral capsule TD0068 and placebo in adults aged 18-65 years with non-severe COVID-19 between September and October 2021. The efficacy outcomes measured included daily cycle threshold (Ct) value from the time of the initial reverse transcription-polymerase chain reaction (RT-PCR) test, time to viral clearance, daily symptom severity score from 15 symptoms of interest, time to symptom resolution, and progression to severe/critical COVID-19. Safety outcomes included adverse events (AEs) and serious adverse events (SAEs).

Results

Sixty patients were randomized (31 received TD0068, and 29 received a placebo). The two groups were balanced in baseline characteristics: mean age was 39 years, and female was predominant (66%). Daily Ct value (median on days 3, 5, 7, and 9 was 25.7, 30.8, 35.4, and 37.6 in the TD0068 group, and 26.4, 31.2, 36.0, and 37.4 in the placebo group, respectively) and time to viral clearance (median: 10 vs. 11 days in TD0068 and placebo groups) were similar between groups. Daily symptom severity score (median on days 3, 5, 7, and 9 was 2, 2, 1, and 0 in the TD0068 group, and 3, 2, 1, and 1 in the placebo group), and time to symptom resolution (median: seven vs. nine days, respectively) were also comparable between groups. No SAE occurred in the study.

Conclusions

TD0068 is safe but does not show an effect for non-severe COVID-19 patients. Further research is needed to explore the potential benefits of garlic in other forms or dosages for the treatment of COVID-19.

## Introduction

The coronavirus disease 2019 (COVID-19) pandemic is still ongoing, with over 660 million people infected worldwide, and nearly 6.7 million deaths have been reported as of January 12, 2023 [1]. More than 13 billion vaccine doses have been administered, and the vaccination has shown a substantial reduction in the number of hospitalizations and severe cases all over the world [1]. However, even with full vaccination plus some boosters, the duration of protection and efficacy of current vaccines against new severe acute respiratory syndrome coronavirus 2 (SARS-CoV-2) variants are uncertain. Currently, the omicron variant predominates. It seems to cause less severe diseases compared to the delta variant, but the transmission is more rapid. When an outbreak occurs, the rapid increase of infected patients can overwhelm the health system, particularly in low-to-middle-income countries (LMICs). Until now, many drugs and treatments have been investigated in the treatment of COVID-19, with more than 5000 trials registered [2]. However, only a few treatments have been recommended by the World Health Organization (WHO), such as systemic corticosteroid, interleukin-6 receptor blockers, baricitinib, casirivimab-imdevimab, nirmatrelvir-ritonavir, molnupiravir, sotrovimab, and remdesivir [3]. However, the recommendations have been changed when there is new evidence, and it is not easy to access these treatments, especially in LMICs. Therefore, there remains the need for other effective treatments for COVID-19.

Previously, a hard-capsule Vietnamese herbal medicine named Kovir (TD0069) has shown safety and efficacy in the treatment of adults with non-severe COVID-19 [4]. It is developed from the Ren Shen Bai Du San remedy and consists of 12 herbal ingredients. The main effect of Kovir is symptom relief and prevention of disease progression. Besides Kovir, there are many other traditional formulae to treat respiratory diseases. Among those, garlic is a well-known herb that has many effects regarding the prevention and treatment of various respiratory diseases [5]. Garlic and its derivatives have demonstrated a significant role in the treatment of COVID-19 [6,7]. Therefore, a traditional medicine (TD0068) was developed from garlic to support the treatment of COVID-19 patients in Vietnam. This study was conducted to assess the safety and efficacy of TD0068 for patients with non-severe COVID-19 in terms of preventing disease progression to severe or critical COVID-19, symptom resolution, and viral clearance.

## Materials and methods

Study design

This study was a phase-II, double-blind, randomized, placebo-controlled trial to evaluate the safety and efficacy of oral hard capsule TD0068 in adults (aged 18-65 years) admitted to the National Hospital for Tropical Diseases, Hanoi, Vietnam, with non-severe COVID-19 between September and October 2021. At this time, all COVID-19 patients had to be quarantined in a hospital. The study was approved by the Ethics Committee of the National Hospital for Tropical Diseases (approval number: 08/HĐĐĐ-NĐTƯ, dated June 8, 2021). The study was done following the principles of the Declaration of Helsinki and the International Conference on Harmonization-Good Clinical Practice guidelines. The protocol was registered with ClinicalTrials.gov (registration number: NCT05082727).

Inclusion and exclusion criteria

Inclusion criteria included: (i) male or female aged 18-65 years, (ii) confirmed SARS-CoV-2 infection by a positive reverse transcription polymerase chain reaction (RT-PCR) test, and (iii) having a non-severe disease with signs and symptoms of COVID-19.

Exclusion criteria included: (i) viral load of <10,000 or a cycle threshold (Ct) value of >30, (ii) having any sign or symptom suggesting severe flu or respiratory infection according to the WHO [8], (iii) acute respiratory distress (partial pressure of oxygen (PaO_2_) < 60 mmHg and/or partial pressure of arterial carbon dioxide (PaCO_2_) > 50 mmHg in room air), (iv) allergy or intolerance to any ingredient of the study drug, (v) inability to swallow the study drug, and (vi) inability to comply with study drug administration or study procedures as judged by investigators.

Study products

TD0068 was developed from a traditional formula called "Thăng ma cát căn thang". Each 515 mg hard capsule consists of 270 mg *Allium sativum* (garlic) extract, 156.4 mg bovine colostrum, 43.6 mg fine powder of mixed herbal medicines, and excipient. The powder of herbal medicines was extracted from seven ingredients: *Rhizoma Cimicifuga foetida*, *Radix Paeoniae Alba*, *Radix Glycyrrhiza uralensis*, *Radix Puerariae*, *Flos Lonicerae*, *Radix Scrophulariae*, and *Fructus Forsythiae*. A placebo was developed with the same shape, size, color, and smell as TD0068.

We carefully chose these ingredients for the development of TD0068, considering their well-documented effects and alignment with traditional medicine principles for herbal remedies. The original formula has a history of use in treating conditions such as colds, headaches, pyrexia, and measles. Garlic, a renowned herb, is known for its efficacy in treating respiratory ailments, including viral infections. Colostrum, with its high levels of antibodies and antioxidants, helps bolster the immune system. Additional ingredients were incorporated to enhance and harmonize the primary effects of garlic extract and colostrum, expediting the body's recovery process.

The raw materials used in the production of TD0068 were collected from specific areas where herbal substances are grown and met the standards set by Vietnam's pharmacopeia before undergoing the extraction process. The herbal extracts used were primarily water extracts, while the garlic was extracted using soybean oil. The preparation of TD0068 was carried out at the branch of Sao Thai Duong Joint Stock Company in Ha Nam, Vietnam, a facility that adheres to the requirements of Good Manufacturing Practice (GMP) for herbal medicine manufacturing.

Randomization and blinding

Eligible patients were randomly assigned to receive either TD0068 or the placebo in a 1:1 ratio. SAS software version 9.4 (Released 2013; SAS Institute Inc., Cary, North Carolina, United States) was used to create block randomization with a block size of four. The randomization codes were kept in envelopes with ordered numbers. All envelopes of a block were kept in another envelope with the block number. Randomized participants were allocated to a treatment group based on the sequential order of recruitment which was the same as the ordered numbers in the envelopes. Investigators, patients, and drug distributors did not know the true study drug of each randomization code.

Study procedures

Right after randomization, participants received either TD0068 hard capsule or a placebo (five tablets x three times per day, i.e., 15 tablets daily in total) for up to 14 days plus standard treatment for COVID-19 patients. The selection of this dosage for TD0068 was informed by three pre-clinical studies, a phase I-II trial involving patients with acute respiratory viral infection, and a phase IIA trial conducted in COVID-19 patients (unpublished data, detailed in Table 1). These studies collectively demonstrated the favorable safety and potential efficacy profile associated with the chosen dosage of TD0068 for the treatment of COVID-19. The standard care included antipyretics, adequate nutrition and rehydration, and other symptomatic treatments according to the WHO guidelines [8].

**Table 1:** Details of unpublished studies that were used as support material for this study COVID-19: coronavirus disease 2019

Type	Author name	Title	Date
Report (at Ha Noi Medicine University, Vietnam)	Nguyen Tran Giang Huong, Dinh Thi Hang, Mai Phuong Thanh, Nguyen Phuong Thanh (Pharmacological Department - Ha Noi Medicine University)	Research Results of Immunostimulatory Effect of Kovir (TD0068) on Cyclophosphamide-Induced Immunodeficiency on Mice	May 9, 2016
Report (at Ha Noi Medicine University, Vietnam)	Nguyen Tran Giang Huong, Dinh Thi Hang, Mai Phuong Thanh, Nguyen Phuong Thanh (Pharmacological Department - Ha Noi Medicine University)	Studies on Acute and Subchronic Toxicity of Kovir (TD0068)	March 3, 2016
Report (at the Ministry of Health, Vietnam)	Nguyen Thanh Binh, Nguyen Thi Hoang Hai (Thai Binh University of Medicine and Pharmacy)	A Phase I/II in Combination To Determine the Maximum Tolerated Dose, Safety, and Dose-Immune Response Relationship of Kovir in Patients With Acute Respiratory Infections Caused by Viruses	March 15, 2019 (Approval certificate No. 28/CN-HĐĐĐ dated March 15, 2019 for phase I)
Report (at the Ministry of Health, Vietnam)	Nguyen Thanh Binh, Nguyen Thi Hoang Hai (Thai Binh University of Medicine and Pharmacy)	A Phase I/II in Combination To Determine the Maximum Tolerated Dose, Safety, and Dose-Immune Response Relationship of Kovir in Patients With Acute Respiratory Infections Caused by Viruses	October 6, 2022 (Approval certificate No. 27/CN-K2ĐT dated October 6, 2022 for phase II)
Report (at the Ministry of Health, Vietnam)	Tran Van Giang (National Hospital for Tropical Diseases)	A Phase IIA, Randomised, Double-Blind, Placebo-Controlled Study To Evaluate the Safety and Effect on Viral Dynamics of TD0068 in the Combination Regimen With Background Treatment in COVID-19 Patients	April 13, 2021 (Approval certificate No. 17/CN-K2ĐT dated April 13, 2021)

Study physicians assessed the patients once daily up to 14 days after randomization to collect data on symptoms’ severity and safety. There were 15 symptoms of interest: fever, headache, chill, sweating, fatigue, muscle pain, sleepiness, nasal secretion, nasal congestion, sneeze, sore throat, hoarseness, cough, chest pain, and loss of smell and/or taste. Each symptom was graded as follows: 0 = absence of the corresponding symptom, 1 = mild, 2 = moderate, and 3 = severe. The daily symptom score was the total score of all 15 symptoms in a day. Daily vital signs, including heart rate, blood pressure, and SpO_2_, were also recorded to monitor safety. Routine laboratory tests, including liver enzymes (alanine aminotransferase (ALT), aspartate aminotransferase (AST)), urea, and creatinine, were done at baseline (before randomization) and once between days 4 and 8.

Semi-quantitative RT-PCR test was performed daily until discharge from nasopharyngeal swab specimens. The test was done at the laboratory of the National Hospital for Tropical Diseases. Viral RNA was extracted using the QIAGEN RNA mini kit (RNeasy® Mini Kit; Qiagen, Hilden, Germany), and PCR targeting the E gene was performed. Extracted nucleic acids were tested for SARS-CoV-2 using RT-PCR with SuperScript™ One-Step RT-PCR System (Thermo Fisher Scientific Inc., Waltham, Massachusetts, United States) on a LightCycler® 480 Instrument, a real-time PCR system (F. Hoffmann-La Roche AG, Basel, Switzerland) in accordance with the manufacturer’s instructions. A quantity of 5 ul of extracted DNA was added to 20 ul of the reaction mix to detect and semi-quantify by one-step RT-PCR using specific primers and probe targeted the E gene of SARS-COV-2 followed strictly the protocol given by the WHO in 2020 [9]. Reactions were incubated at 50°C for 10 minutes and 95°C for five minutes, followed by 45 cycles at 95°C for 10 seconds and 58°C for 40 seconds. For quality control, two negative controls, one positive control, and one internal control were included in each PCR batch to avoid any false results. The limit of detection of this procedure was 5.2 copies/reaction.

Criteria for hospital discharge were in accordance with the guidance of the Vietnamese Ministry of Health, which included: (i) having no fever for at least three days, (ii) improved clinical symptoms and chest X-ray findings, and normal vital signs and routine blood tests, and (iii) three negative RT-PCR results on three consecutive specimens taken at least one day apart.

Study endpoints

The efficacy outcomes included daily Ct value, time to viral clearance, seven- and 14-day viral clearance, daily symptom severity score, time to symptom resolution, and the progression to severe or critical COVID-19. Time to viral clearance was the number of days from randomization to the first day that viral RNA was undetectable (i.e., Ct value was more than 40). Time to symptom resolution was the number of days from randomization to the first day that all symptoms were cleared, i.e., the symptom score was 0. Severe and critical COVID-19 were based on the WHO guidelines [8]: severe COVID-19 was defined when the patients had an oxygen saturation of <90% on room air, signs of pneumonia, or signs of severe respiratory distress; critical COVID-19 included acute respiratory distress syndrome, sepsis, septic shock, and requirement of life-sustaining treatments. Safety outcomes included all adverse events (AEs), serious adverse events (SAEs), and discontinuations of study products due to AEs.

Statistical analysis

A convenient sample size of 60 participants (30 in each treatment group) was chosen since this is the first study to investigate the safety and efficacy of TD0068 for non-severe COVID-19 patients.

The intention-to-treat (ITT) principle was applied for efficacy analyses. All randomized participants were included in the analyses, and the patients were analyzed in their randomized treatment group irrespective of the actual study drug that they received and premature discontinuation of the study drug. The safety outcomes were analyzed with an as-treated dataset. Patients were analyzed in their actual treatment groups.

Time-to-event outcomes (time to viral clearance and time to symptom resolution) were summarized and visualized by the Kaplan-Meier method and were compared between the two groups by log-rank test. Daily Ct value and daily symptom severity score were visualized by boxplots and were compared between groups by Wilcoxon Mann-Whitney-U test. Binary outcomes (seven- and 14-day viral clearance, progression to severe or critical COVID-19, and safety outcomes) were summarized by frequency and percentage and were compared by Fisher’s exact test. Since vaccination is a strong protective factor for COVID-19 patients, we performed a subgroup analysis of patients with and without vaccination for time to viral clearance and time to symptom resolution to explore whether the effect of TD0068 differed between these groups. The statistical software R version 4.1.3 (Released 2022; R Foundation for Statistical Computing, Vienna, Austria) was used for all analyses.

## Results

**Figure 1:**
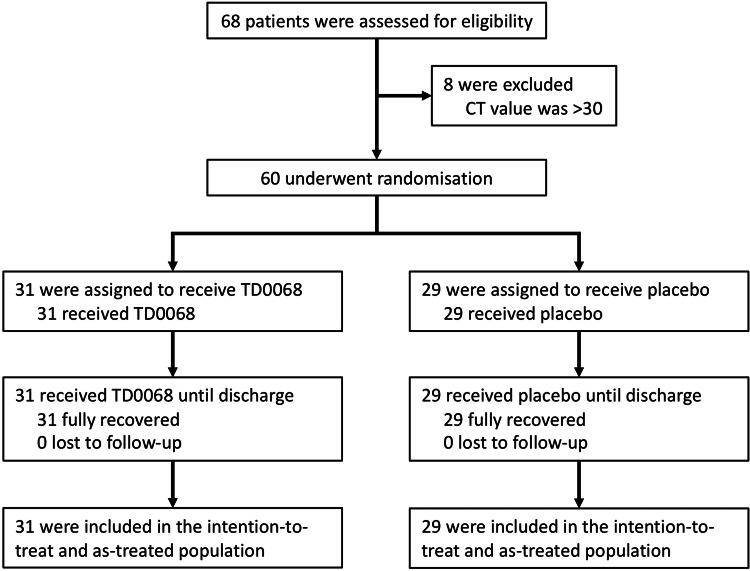
Study flowchart CT: cycle threshold

Between 16 September 2021, and 20 October 2021, 68 patients were screened and eight were excluded because the Ct value was >30. Finally, 60 eligible patients were randomized: 31 were assigned to receive TD0068, and 29 were assigned to receive the placebo. All participants completed their treatment until discharge. Finally, all 60 randomized participants were included in the ITT and as-treated populations (Figure 1).

The two treatment groups were balanced with regard to baseline characteristics (Table 2). The mean age was 38.9 ± 11.4 vs. 39.0 ± 10.5 years in the TD0068 and placebo groups, respectively. The female gender was predominant in both groups (64.5% vs. 69%, respectively). Most patients had a normal body mass index (93.5% vs. 93.1%, respectively). None of the patients were infected by COVID-19 previously; 45.2% of the patients in the TD0068 group and 51.7% in the placebo group received COVID-19 vaccination but most received only one dose. The mean time from symptom onset to recruitment was 3.5 days in both groups. The mean baseline symptom score was 4.8 ± 3.1 vs. 4.4 ± 2.3 in TD0068 and placebo groups, respectively. Baseline Ct value was also similar in both groups: 22.5 ± 4.0 vs. 22.6 ± 4.2, respectively. All routine blood test results were comparable between the two groups.

**Table 2:** Baseline characteristics Summary statistics are mean ± standard deviation, median (25^th^; 75^th^ percentiles), or n (%) BMI, body mass index; COVID-19, coronavirus disease 2019; Ct, cycle threshold.

	TD0068 (N=31)	Placebo (N=29)
Sex female	20 (64.5)	20 (69.0)
Age (years)	38.9 ± 11.4	39.0 ± 10.5
BMI (kg/m^2^)	21.7 ± 1.7	21.5 ± 1.8
Nutritional status		
Underweight (BMI < 18.5 kg/m^2^)	1 (3.2)	1 (3.4)
Normal weight (BMI: 18.5-24.9 kg/m^2^)	29 (93.5)	27 (93.1)
Overweight (BMI: 25-29.9 kg/m^2^)	1 (3.2)	1 (3.4)
Obesity (BMI ≥ 30 kg/m^2^)	0 (0.0)	0 (0.0)
COVID-19 vaccination	14 (45.2)	15 (51.7)
Number of vaccine doses received		
0	17 (54.8)	14 (48.3)
1	9 (29.0)	10 (34.5)
2	5 (16.1)	5 (17.2)
Previous COVID-19 infection	0 (0.0)	0 (0.0)
Comorbidities	2 (6.5)	2 (6.9)
Time from symptom onset to recruitment (days)	3.5 ± 1.2	3.5 ± 1.5
Baseline symptom score	4.8 ± 3.1	4.4 ± 2.3
Baseline Ct value	22.5 ± 4.0	22.6 ± 4.2
Alanine aminotransferase (U/L)	19 (13; 32)	21 (16; 38)
Aspartate aminotransferase (U/L)	25 (22; 28)	28 (22; 34)
Urea (mmol/L)	4.2 ± 1.0	4.4 ± 1.2
Creatinine (umol/L)	69.4 ± 12.7	70.4 ± 13.6
White blood cell count (x 10^9^/L)	6.3 ± 2.4	6.2 ± 2.4
Neutrophil count (x 10^9^/L)	4.0 ± 2.2	4.0 ± 1.9
Lymphocyte count (x 10^9^/L)	1.5 ± 0.7	1.5 ± 0.7

The number of study products used was balanced between groups: median (interquartile range) was 42 (34; 42) and 42 (33; 42) in TD0068 and placebo groups, respectively. Other treatments were also similar in the two groups. The most common concomitant drug was cough suppressants (90.3% vs. 86.2% in TD0068 and placebo groups, respectively), followed by anticoagulants (22.6% vs. 34.5%) and antipyretics (22.6% vs. 17.2%). Antibiotics, antivirals, and corticosteroids were rarely used. The median hospital length of stay was 14 days in both groups (Table 3).

**Table 3:** Study products administration and concomitant treatments Summary statistics are median (25^th^; 75^th^ percentiles) or n (%).

	TD0068 (N=31)	Placebo (N=29)	P-value
Total number of doses	42 (34; 42)	42 (33; 42)	0.731
Cough suppressants	28 (90.3)	25 (86.2)	0.702
Anticoagulants	7 (22.6)	10 (34.5)	0.394
Antipyretics	7 (22.6)	5 (17.2)	0.750
Antibiotics	3 (9.7)	2 (6.9)	1
Antivirals	1 (3.2)	0 (0.0)	1
Corticosteroids	1 (3.2)	0 (0.0)	1
Hospital length of stay (days)	14 (12; 14)	14 (12; 15)	0.541

Eight patients experienced any AE, including two (6.5%) in the TD0068 group and six (20.7%) in the placebo group. All AEs were evaluated as mild and unrelated to the study drugs. No SAE and no study product discontinuation due to AE occurred (Table 4).

**Table 4:** Safety and efficacy outcomes Summary statistics are n (%). COVID-19, coronavirus disease 2019.

	TD0068 (N=31)	Placebo (N=29)	P-value
Any adverse event	2 (6.5)	6 (20.7)	0.140
Type of adverse event			-
Elevated D-dimer	0 (0.0)	1 (16.7)	
Elevated liver enzymes	2 (100.0)	3 (50.0)	
Diarrhea	0 (0.0)	2 (33.3)	
Serious adverse event	0 (0.0)	0 (0.0)	-
Premature discontinuation of study products due to adverse events	0 (0.0)	0 (0.0)	-
Viral clearance at day 7	2 (6.5)	3 (10.3)	0.666
Viral clearance at day 14	24 (77.4)	22 (75.9)	1
Progression to severe/critical COVID-19	0 (0.0)	0 (0.0)	-

**Figure 2:**
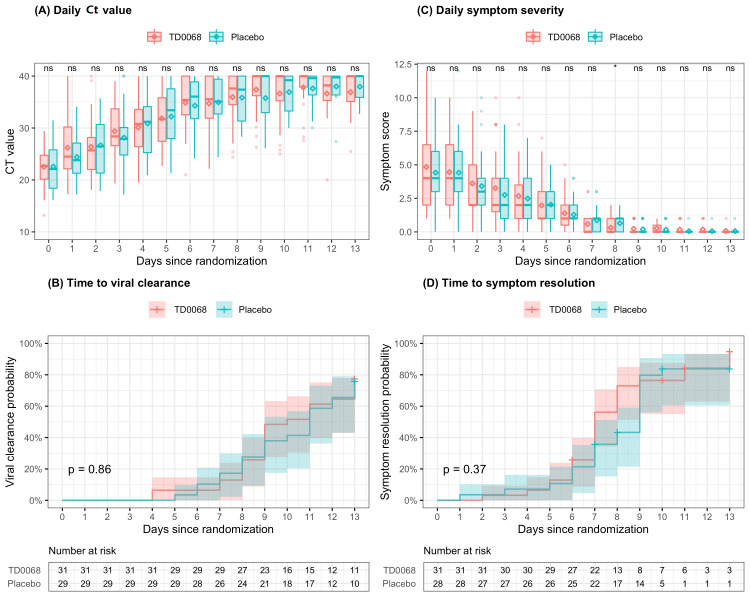
Viral clearance and symptom resolution In (A) and (C), the line inside each box is the median, the upper and lower margins of each box are the 25th and 75th percentiles, and the diamond inside each box is the mean of symptom score. Mann-Whitney-U test is used to compare symptom scores between the two groups and symbols indicating statistical significance are presented in the top of each plot: **** < 0.0001 < *** < 0.001 < ** < 0.01 < * < 0.05 < ns. In (B) and (D), lines are the Kaplan-Meier estimates and colored regions are 95% confidence intervals. In (D), one patient in placebo group was excluded from the analysis because this patient had symptom score of 0 at randomization. ns, not significant; Ct, cycle threshold

Ct value increased over time and was not significantly different between TD0068 and placebo groups on any day since randomization (Figure 2A). Therefore, the time to viral clearance was similar between the two groups (Figure 2B). The median time to viral clearance was 10 days and 11 days in TD0068 and placebo groups, respectively. The proportion of viral clearance on days 7 and 14 was also similar in the two groups: 6.5% vs. 10.3%, and 77.4% vs. 75.9%, respectively. No patient experienced the progression to severe or critical COVID-19 (Table 4). Viral clearance time was unknown in 21 patients (11 in the TD0068 group and 10 in the placebo group). They all had negative PCR test after day 14.

Symptom severity score decreased over time and was comparable between the two groups (Figure 2C). Most patients cleared all symptoms after day 8. Time to symptom resolution was similar between the groups (Figure 2D). Median time to symptom resolution was seven days vs. nine days in TD0068 and placebo groups. A patient in the placebo group had none of the 15 symptoms of interest. Therefore, the symptom score was 0 since randomization, and this patient was excluded from the Kaplan-Meier estimate.

**Figure 3:**
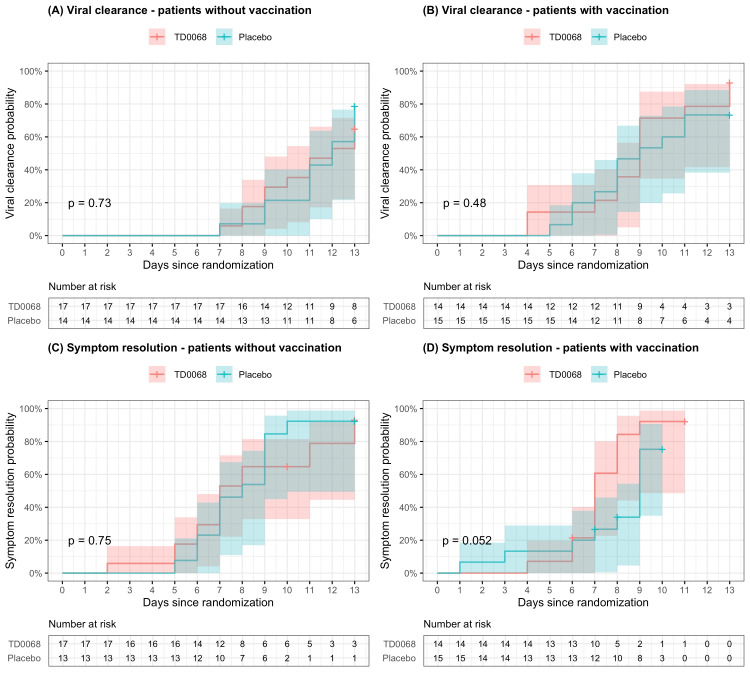
Viral clearance and symptom resolution – subgroup analysis by patients with and without vaccination Lines are the Kaplan-Meier estimates and colored regions are 95% confidence intervals.

The subgroup analysis resulted in similar results between patients with and without vaccination. There was no statistically significant difference between TD0068 and placebo groups in time to viral clearance and time to symptom resolution in patients with and without COVID-19 vaccination (Figure 3).

## Discussion

This study evaluated the safety and efficacy of TD0068, a Vietnamese herbal medicine extracted from garlic combined with other herbal ingredients, in the treatment of non-severe COVID-19 adults. We found that TD0068 was safe. However, TD0068 did not show efficacy when compared with the placebo regarding viral clearance and symptom resolution.

Many herbal and traditional medicines have been studied for the treatment of COVID-19, including non-severe and severe/critical diseases [4,10-25]. These medicines were developed from a wide range of herbs and formulae from countries with well-known traditional medicine, such as China, Iran, India, and Vietnam. In general, a prominent characteristic of herbal medicines is symptom relief and prevention of disease progression. Few studies showed the effect of reducing SARS-CoV-2 viral load, but the evidence needs to be strengthened. The association between viral load and COVID-19 severity is unclear [26,27]. Therefore, a treatment could improve the clinical outcomes of COVID-19 patients without reducing the viral load. Currently, most treatments recommended by the WHO to treat COVID-19 patients affect the host immune system rather than the virus. This might be the vital issue of severe COVID-19, particularly in the late stages.

The study product was developed mainly from garlic (*Allium sativum L.*). Recent publications have reported conflicting results regarding the efficacy of garlic extracts in treating COVID-19 [28,29]. Specifically, an observational study on garlic essential oil demonstrated significant effectiveness [28], while a clinical trial on fortified garlic extract oral capsules showed no significant effect in treating COVID-19 [29]. Our study's findings are consistent with the results of the latter clinical trial, indicating no effect of garlic products on COVID-19 patients. The variations in results could potentially be attributed to differences in product form, components, study design, and participant population.

However, some laboratory studies have shown the effect of garlic in the inhibition of SARS-CoV-2 replication [30-34]. In our study, garlic extract was combined with bovine colostrum and other herbal ingredients. Bovine colostrum has shown potential benefits for human health with some bioactive proteins such as immunoglobulins, lactoferrin, lactoperoxidase, and oligosaccharides [35,36]. Despite this, our study did not find any significant effect of this combination in treating non-severe COVID-19 patients in terms of viral clearance and symptom resolution. This finding was consistent in patients with and without vaccination. Determining the specific reasons why TD0068 did not exhibit an effect in COVID-19 treatment is challenging. Possible factors to consider include the composition of garlic extracts, the combinations with colostrum and other herbs, the dosage used, and other aspects that warrant further investigation. While garlic may still have potential benefits for COVID-19 patients, it should be explored in alternative forms or combinations. Additional laboratory and clinical studies are necessary to comprehensively examine the role of garlic and garlic extracts in the context of COVID-19.

This study has several limitations. First, this is an exploratory trial with a small sample size. Consequently, there is a possibility of biases or confounding factors that may have influenced the results. The study does not provide adequately robust evidence to eliminate the efficacy of TD0068. However, the findings do not support further investigation of this product for patients with COVID-19. Second, most participants were young, had a normal body mass index, and had no comorbidities. The study population does not represent the whole population, particularly the higher-risk patients with comorbidities, which limits the generalizability of the findings. Third, due to limited resources, we did not investigate inflammatory biomarkers in this study. The effect of TD0068 on the inflammatory response of the patients, therefore could not be found.

## Conclusions

The Vietnamese medicine (TD0068) developed mainly from garlic is safe for patients with non-severe COVID-19. However, the study did not show an effect of TD0068 in terms of viral clearance and symptom resolution. Further research is needed to explore the potential benefits of garlic in other forms or dosages for the treatment of COVID-19.
